# *Pocillopora* spp. growth analysis on restoration structures in an Eastern Tropical Pacific upwelling area

**DOI:** 10.7717/peerj.13248

**Published:** 2022-06-23

**Authors:** Lisa Combillet, Sònia Fabregat-Malé, Sebastián Mena, José Andrés Marín-Moraga, Monica Gutierrez, Juan José Alvarado

**Affiliations:** 1 Master Sciences pour l’Environnement, parcours Gestion de l’environnement et écologie Littorale, Université de La Rochelle, La Rochelle, France; 2 Posgrado en Biología, Sistema de Estudios de Posgrado, Universidad de Costa Rica, San Pedro de Montes de Oca, San José, Costa Rica; 3 Escuela de Biología, Universidad de Costa Rica, San Pedro de Montes de Oca, San José, Costa Rica; 4 Raising Coral Costa Rica, San José, Costa Rica; 5 Sostenibilidad, Península Papagayo, Guanacaste, Costa Rica; 6 Centro de Investigación en Biodiversidad y Ecología Tropical (CIBET) (Previously Museo de Zoología), Universidad de Costa Rica, San Pedro de Montes de Oca, San José, Costa Rica; 7 Centro de Investigación en Ciencias del Mar y Limnología (CIMAR), Universidad de Costa Rica, San Pedro de Montes de Oca, Costa Rica

**Keywords:** Branching coral, Fragmentation, Growth rates, Artificial structure, Coral gardening

## Abstract

Coral reefs in Culebra Bay (North Pacific of Costa Rica) are threatened by multiple anthropogenic disturbances including global warming, overfishing, eutrophication, and invasive species outbreaks. It is possible to assist their recovery by implementing ecological restoration techniques. This study used artificial hexagonal steel structures, called “spiders” to compare growth of *Pocillopora* spp. coral fragments of different sizes. Three initial fragment class sizes were used: 2, 5 and 8 cm, with each class size having 42 initial fragments. Changes in fragment length, width and area were measured monthly from January to December 2020. Results showed an overall survivorship of 70.21%, and no significant differences in survivorship and linear growth rate were detected between class sizes. The linear growth rates are 4.49 ± 1.19 cm year^−1^, 5.35 ± 1.48 cm year^−1^ and 3.25 ± 2.22 cm year^−1^ for the 2, 5 and 8 cm initial class sizes, respectively. Our results do not show significant differences in growth rates between the different initial fragment sizes. However, since small fragments (2 cm) present higher mortality during the first month, we recommend using larger fragments. In addition, coral fragments grew 48% more during the non-upwelling season, which may suggest that it might be more effective and safer to start the restoration efforts during this period.

## Introduction

Coral reefs are highly diverse ecosystems that provide essential goods and services to hundreds of millions of people (Knowlton et al., 2021), such as food, livelihoods through fisheries and tourism, protection from coastal erosion and storms, and cultural practices (Woodhead et al., 2019). Nevertheless, in the last decades, many reefs around the world have collapsed, and live coral cover has declined due to several factors, such as climate change, acidification and unplanned coastal development (Hughes et al., 2017; El-Naggar, 2020; Knowlton et al., 2021). The rapid deterioration of these ecosystems threatens the stability of marine environments and human well-being (Eddy et al., 2021).

Due to this intense degradation of coral reefs worldwide and in the face of future climate change, ecological restoration of coral reefs is becoming an increasingly important management approach (McLeod et al., 2021). Restoration of degraded coral reefs can be achieved through different means, using either sexual or asexual coral recruits in order to enhance coral populations (Rinkevich, 1995; Rinkevich, 2019). During the last 20 years, several restoration techniques have been developed, and coral gardening has been one of the most widely used. This approach is based on the asexual propagation of corals by the fragmentation of wild donor colonies. The collected fragments are later put into coral nurseries, where they grow until they become larger colonies which are later outplanted onto a degraded reef (Rinkevich, 2006). A wide variety of structures have been used as coral nurseries, from floating (suspended in the water column) to fixed structures (on the seafloor) (Shafir & Rinkevich, 2010; Rinkevich, 2019).

Most restoration projects have been developed in the Caribbean and Indo-Pacific (Boström-Einarsson et al., 2020). In the Eastern Tropical Pacific (ETP), however, coral reef restoration is still in its infancy, and very few projects are based on coral gardening (Bayraktarov et al., 2020). Conditions in the ETP are different from those in the Caribbean and Indo-Pacific. Coral reefs are relatively small (a few hectares), discontinuous, and are built by few coral species, predominantly of the genera *Pocillopora*, *Porites* and *Pavona* (Guzmán & Cortés, 1993; Glynn et al., 2017). The region comprises three seasonal upwelling areas (Gulf of Tehuantepec, Gulf of Papagayo and Gulf of Panama), with incursions of deep, cold and nutrient-rich waters (Cortés, 1997; Fiedler & Lavín, 2017). The ETP is also affected by the El Niño-Southern Oscillation (ENSO), which causes an increase in sea surface temperatures that can lead to coral bleaching and high mortality, with loss of live coral cover (Glynn, 1984; Guzmán et al., 1987; Jiménez et al., 2001; Jiménez & Cortés, 2001; Brainard et al., 2018).

**Figure 1:**
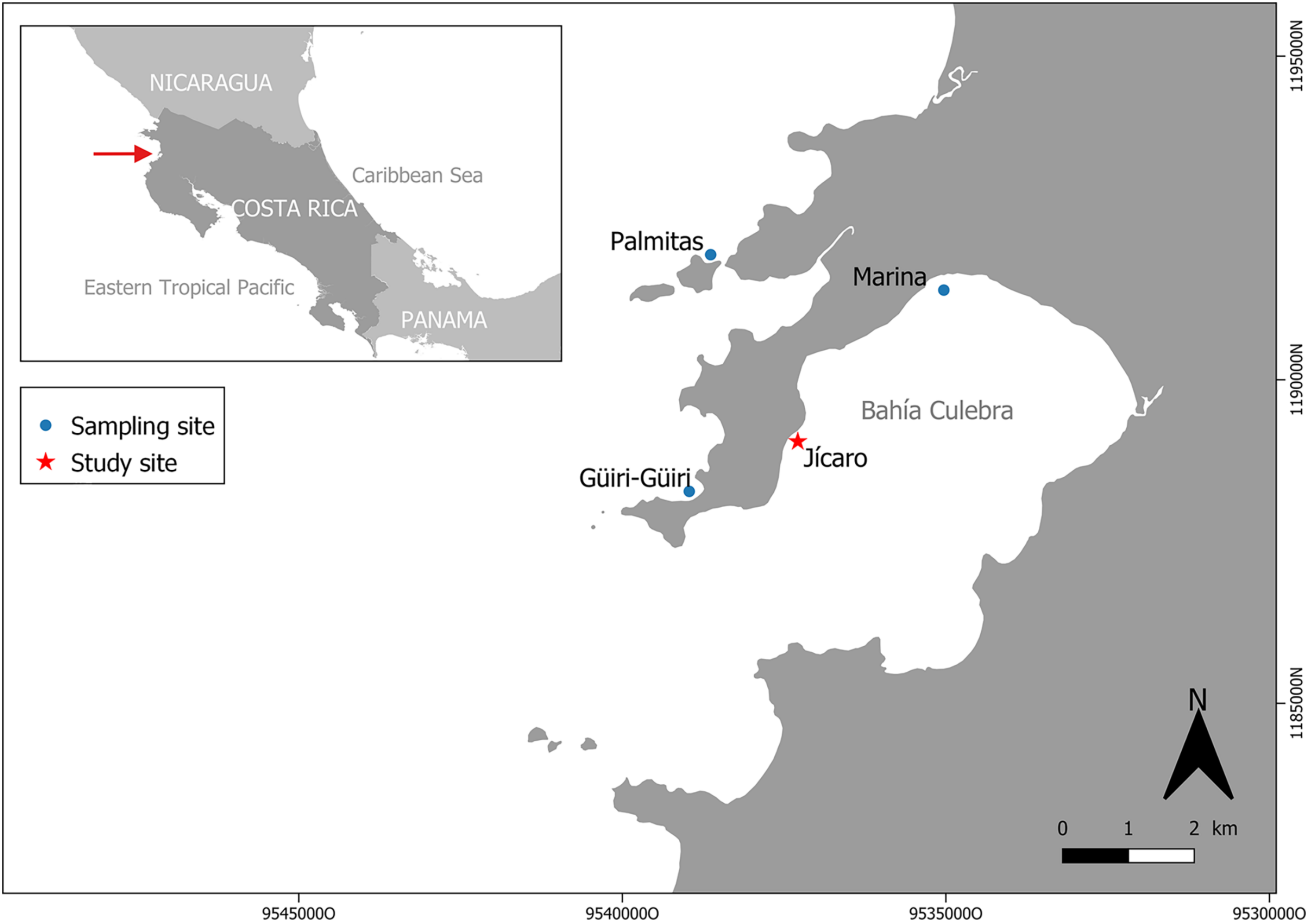
Study and sampling site in Culebra Bay, North Pacific of Costa Rica.

The North Pacific coast of Costa Rica was considered as one of the best regions for the development of coral reefs in the country (Cortés & Jiménez, 2003; Alvarado et al., 2018). Within it, the reefs in Culebra Bay (Fig. 1) were considered as the most diverse, but in the last two decades various disturbances caused severe degradation that led to the collapse and loss of many reefs around the bay. Red tides and macroalgal proliferation induced coral bleaching and mortality (Cortés et al., 2010). The following increase in sea urchin populations (*Diadema mexicanum*) resulted in high bioerosion rates and caused the loss of the reefs’ structural complexity and framework (Alvarado, Cortés & Reyes-Bonilla, 2012; Alvarado et al., 2016), which in turn had an impact on diversity of reef-associated organisms and ecosystem functions (Arias-Godinez et al., 2019; Salas-Moya et al., 2021).

The particular environmental conditions, combined with the relatively low experience on coral reef restoration in the region, means that little is known about restoration techniques and specific considerations about the species used. However, some studies (mostly in Mexico and Colombia) have been carried out using the coral genus *Pocillopora* (Liñan-Cabello et al., 2011; Tortolero-Langarica, Cupul-Magaña & Rodriguez-Troncoso, 2014; Nava & Figueroa-Camacho, 2017; Lizcano-Sandoval, Londoño-Cruz & Zapata, 2018; Ishida-Catañeda et al., 2020; Vargas-Ugalde et al., 2020). The restoration project implemented in Culebra Bay, which was initiated in 2019, could help determine the optimal initial coral fragment size and the suitability of a new technique and thus, help respond to specific research questions for the development of coral restoration projects in the ETP. Coral fragments of *Pocillopora* spp. of three different initial sizes were attached to the structures, and their growth was monitored monthly for one year. The aim of the present study is to determine whether coral fragment growth and survival is affected by initial fragment size and presence of upwelling, in order to establish the optimal fragment size and best period to start restoration efforts for *Pocillopora* dominated reefs.

## Materials and Methods

### Study area

Culebra Bay, in the Gulf of Papagayo, is located in the Guanacaste province of Costa Rica, in the Northwest Pacific of the country. This bay consists of a series of islets, beaches, cliffs and estuaries with important economic marine resources, and it is subject to a seasonal upwelling between December and April, which brings up colder and nutrient-rich waters (Jiménez, 2001; Alfaro & Cortés, 2012). During this period, seawater temperatures can decrease by 8 to 9 °C from the annual average (27.9 °C) (Alfaro & Cortés, 2012; Alfaro et al., 2012). The bay is naturally exposed to lower pH (pH = 7.8) than other regions, with high temporal variability following the dry and rainy seasons (Sánchez-Noguera, Jiménez & Cortés, 2018). Even during the non-upwelling season (from May to November) there is a reduced pH that impacts photosynthesis, respiration, and calcification processes (Rixen, Jiménez & Cortés, 2012; Sánchez-Noguera, Jiménez & Cortés, 2018). Sedimentation in the area is low (3.0 ± 0.78 mg cm^−2^ day; Fernández-García et al., 2012) and without any sign of human stress (Rogers, 1990). Coral reefs in the bay are dominated by the genus *Pocillopora*, which forms monospecific patches that used to cover several hectares along the bay in the 1990s. On some reefs, corals covered between 40% and 80% of the substrate (Jiménez, 2001; Cortés & Jiménez, 2003). In 2010, however, live coral cover was only 1% to 4% (Sánchez-Noguera et al., 2018). The main *Pocillopora* species in the region are *Pocillopora damicornis* (Linnaeus, 1758) and *Pocillorpora elegans* (Dana, 1846). In this study, these two species were grouped under the name of *Pocillopora* spp. because the morphologies are similar, with intermediate shapes, which make their precise identification in the field difficult. The experiment took place in the coral reef patch in front of Playa Jícaro (10.619830°N, 85.675810°W) (Fig. 1).

### Experimental design

**Figure 2:**
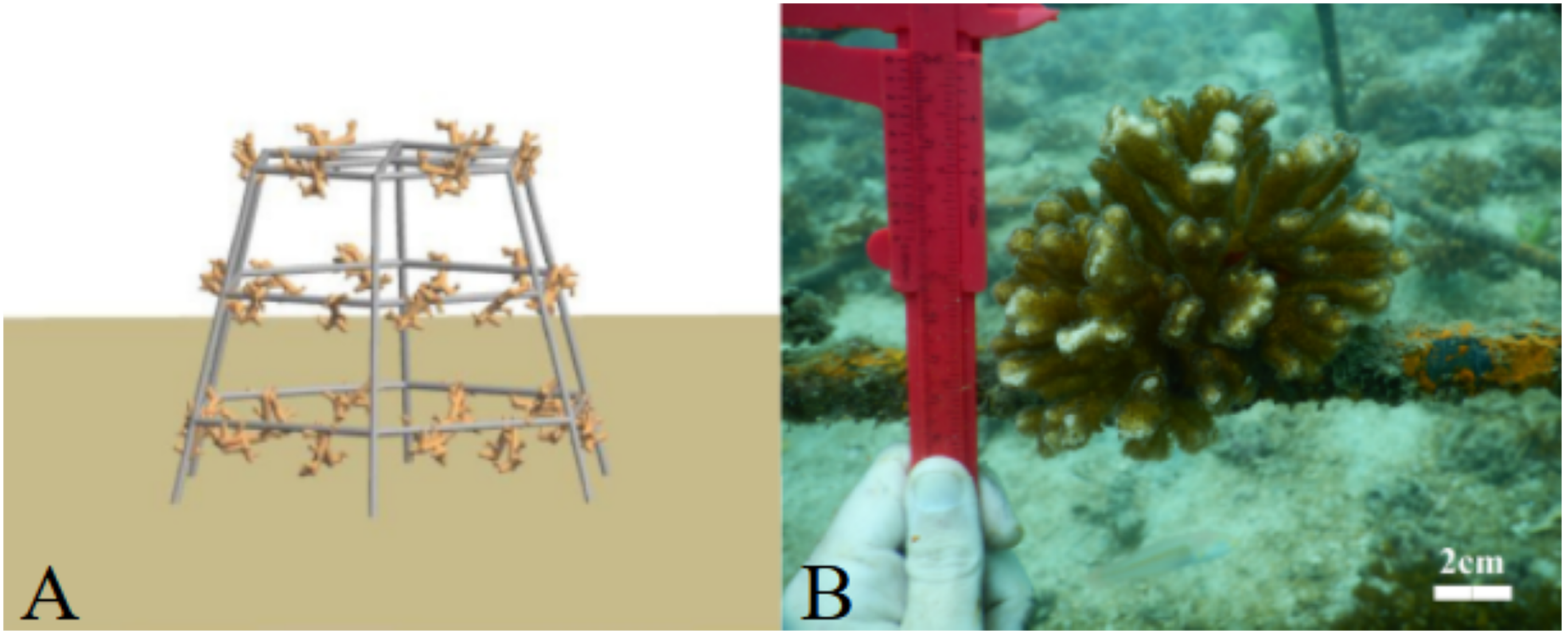
“Spider” structures used for coral restoration in Playa Jícaro, Culebra Bay, North Pacific of Costa Rica. Side view of the “spider” structure (A) and method used to monitor *Pocillopora* coral fragments attached to the structure (B).

*Pocillopora* spp. fragments (*n* = 126) were obtained from colonies on three sites around Culebra Bay: Palmitas, Marina and Güiri-Güiri (Fig. 1). Healthy large donor colonies (>30 cm in diameter and without observable injuries) were randomly selected at depths between 3 to 8 m, and no more than three fragments were obtained from each donor. Three different initial fragment sizes categories were considered: small (2 cm, 2.57 ± 0.38 cm), medium (5 cm, 5.35 ± 0.78 cm) and large (8 cm, 8.26 ± 1.63 cm). Forty-two fragments from each size class were attached using plastic cable ties to three “spider” restoration structures, one for each fragment size class. These hexagonal metallic structures are 90 cm high and have three levels, 25, 35 and 45 cm long from top to bottom, and 30 cm apart. Arrangement of the coral fragments within the structure (for each of the six sides of the “spider”, two fragments on the top level, two in the middle and three in the lowest) was based on fragments having enough space to grow and not competing with each other (Fig. 2A). This design also allows coral fragments to grow on the external side of the structure and if they break and fall from it, they can continue to grow surrounded by other fragments on the seafloor, forming a three-dimensional structure. With this method, corals are not necessarily destined to be outplanted to the reef afterwards, but to stay on the structures, where they can keep growing. Thus, “spiders” have a double purpose, as they can act as both a nursery and a substrate on which to permanently attach corals to contribute to the structural complexity of the reef. The three “spiders” were placed at 6 m depth, on the front reef area.

### Data collection

The experiment was conducted from January to December 2020. March 2020 is excluded from the results because of the COVID-19 sanitary crisis, which prevented data collection in Costa Rica. The study site was visited monthly and each *Pocillopora* fragment was photographed with an underwater Nikon COOLPIX W300 camera, using a calliper as a scale (Fig. 2B). Photographs were later analysed using ImageJ software, which allows for a 0.001 cm precision, and height (cm), width (cm) and area (cm^2^) of each fragment was determined. This allowed us to estimate a growth rate in terms of linear extension (cm year^−1^) and tissue area (cm^2^ year^−1^). Linear extension was calculated by measuring the vertical length between the two longest coral branches, while the area of the coral was estimated by outlining the contour of the coral fragment, and subsequently calculating the average. Mortality was visually determined; a fragment was considered dead if it had no living tissue left and/or was covered by other organisms such as algae, barnacles or ascidians. If the fragment was partially dead, only the part with living tissue was measured. The number of dead fragments was established and used to calculate fragment survival rates. Seawater temperature in the restoration area was recorded using HOBO**®** data loggers, which were set to record data every 30 min.

### Data analysis

Survival rates of each initial size class were calculated and compared with a Chi-squared contingency test in order to determine the influence of the initial size of the fragment. Lost fragments were excluded from this calculation since it is not possible to establish whether they survived. Means of fragments length, width, and area at initial time (January 2020) and every month until December 2020 were estimated for each “spider” and then, the relative growth between initial and final time was calculated. These estimations considered only fragments that survived until the last month of the experiment and excluded fragments that broke during the course of the experiment. Means of fragment length, width and area of each month are compared with a one-way ANOVA followed by Tukey HSD post-hoc tests. To compare the absolute growth and growth rate between the three different initial class sizes, a two-way ANOVA test was used followed by a Tukey HSD post-hoc test. Finally, a t-test was used to compare the difference in growth between two periods: from January to April and from May to December, according to the presence and absence of seasonal upwelling, respectively. Monthly average, minimum and maximum seawater temperature was calculated from Hobo data. Statistical analyses were performed using R, including the package “stats” (R Core Team, 2018).

## Results

### Coral fragment survival

At the end of the experiment, 66 fragments survived (52.38%), 28 died (22.22%), and 32 were lost (25.39%) due to fragmentation or cable tie break during the experimentation (Table 1). Excluding lost fragments, coral fragment survival is not affected by the initial fragment size ( $X^2$ = 3.993, df = 2, *p* > 0.05). The overall survival rate from January to December 2020 is 70.21%. The highest number of death fragments (9) appeared in February, whilst the number of dead fragments during other months ranged from 0 to 5. In order to determine whether upwelling had an effect on coral mortality, a Pearson’s chi-squared test was also performed between upwelling season (January to April) and non-upwelling season (May to December). No significant differences in mortality were observed between the two periods ( $X^2$ = 1.5345, df = 1, *p* > 0.05). The test also showed no statistical differences when considering initial fragment size ( $X^2$ = 3.247, df = 2, *p* > 0.05 and $X^2$ = 0.812, df = 2, *p* > 0.05).

**Table 1:** Survival by initial fragment size of *Pocillopora* spp. fragments at the end of the experiment in Jícaro reef, Culebra Bay.

Initial size	Alive	Dead	Lost
A (2 cm)	15 (35.71%)	12 (28.57%)	15 (35.71%)
B (5 cm)	23 (54.76%)	8 (19.05%)	11 (26.19%)
C (8 cm)	28 (66.67%)	8 (19.05%)	6 (14.29%)
Total	66 (52.38%)	28 (22.22%)	32 (25.39%)

**Note:**

Survival is not affected by initial fragment size ( $X^2$ = 3.993, df = 2, *p* > 0.05).

### Coral fragment growth

**Figure 3:**
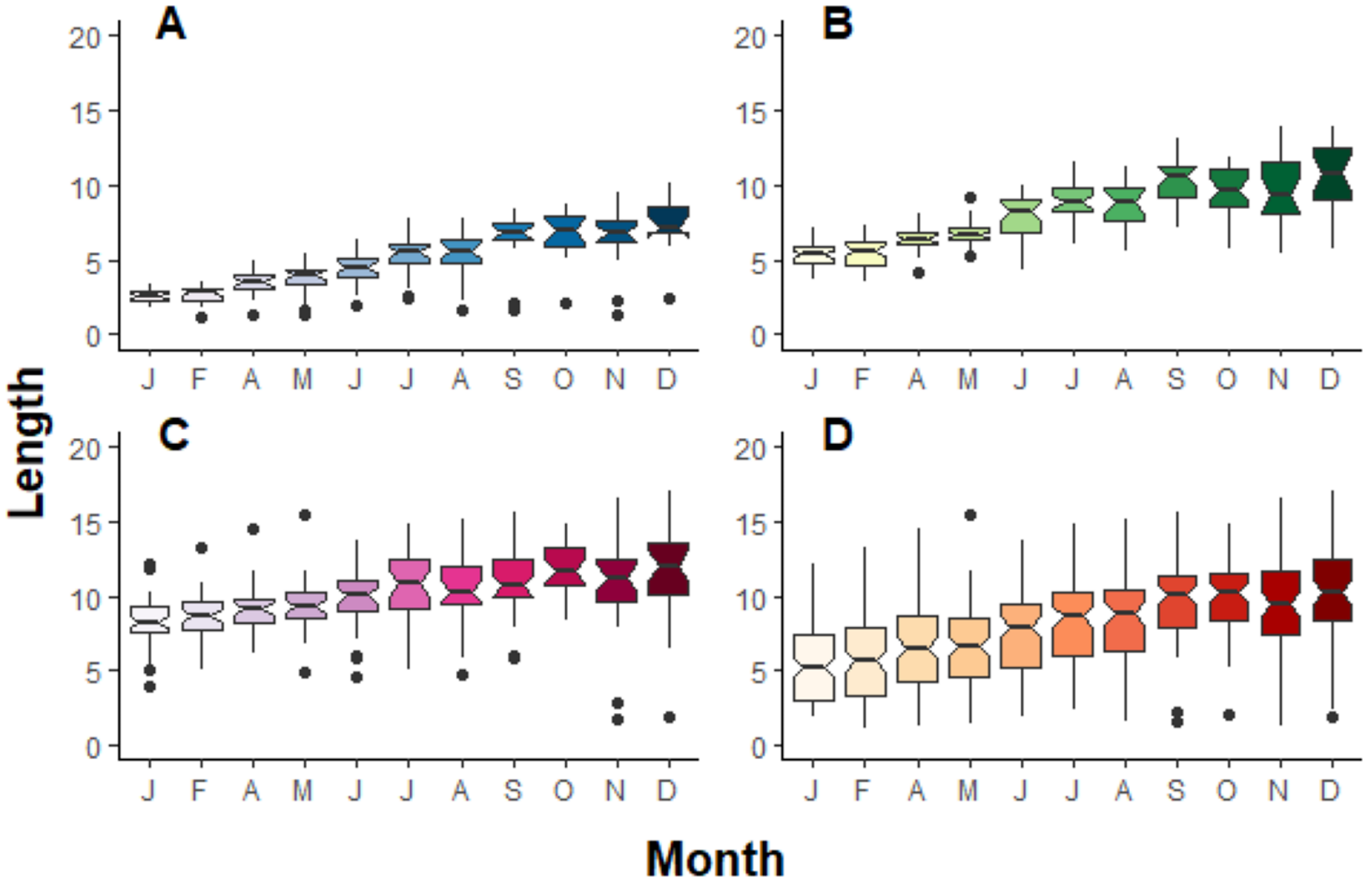
Boxplot compilation of fragment length growth over a year depending on their initial size in Jícaro reef, Culebra Bay (A = 2 cm, B = 5 cm, C = 8 cm, D = all sizes). The ANOVA test followed by the Tukey HSD test shows a significant difference between, at least, January and December for all the initial fragment sizes ((A) *p* < 0.001, F_10,261_ = 39.9; (B) *p* < 0.001, F_10,321_ = 56.5; (C) *p* < 0.001, F_10,349_ = 9.5; (D) *p* < 0.001, F_10,953_ = 35.2).

During the period of observation, *Pocillopora* fragments grew significantly in terms of length (F_10,953_ = 35.2, *p* > 0.001), width (F_10,935_ = 40.8, *p* > 0.001) and area (F_10,953_ = 46.5, *p* > 0.001), independently of their initial class size (Fig. 3). On average, fragments grew 4.12 ± 2.77 cm year^−1^ and quadrupled their surface over one year (438%). *Pocillopora* fragments grew more in terms of length than width (Table 2). For some fragments, negative growth between months was observed. Growth rate in length and width does not significantly differ by initial class size, but it does for area measurements: A is significatively different from B and C, and B is significatively different from C (Table 2).

**Table 2:** Mean growth rate of *Pocillopora* spp. fragments from January to December 2020 depending on their initial class size in Jícaro reef, Culebra Bay.

Initial class size	Length		Width		Area	
	Growth rate (cm year^−1^)	Relative growth (%)	Growth rate (cm year^−1^)	Relative growth (%)	Growth rate (cm^2^ year^−1^)	Relative growth (%)
A	4.49 ± 1.19	164 ± 78	3.38 ± 1.19	222 ± 181	21.08 ± 6.32*	752 ± 493
B	4.28 ± 1.48	92 ± 43	4.28 ± 1.53	162 ± 93	46.36 ±12.67*	526 ± 303
C	3.25 ± 2.22	45 ± 59	3.05 ± 2.15	73 ± 83	36.32 ± 18.73*	189 ± 161
Total average	4.12 ± 2.77	87 ± 74	3.51 ± 2.47	139 ± 130	34.97 ± 23.01	438 ± 384

**Note:**

A = 2 cm, B = 5 cm, C = 8 cm (*represent the significant differences between class sizes). The two-way ANOVA and Tukey HSD tests for the area measures show significant differences between all class sizes (F_4,186_ = 5.49): A and B (*p* < 0.001); A–C (*p* < 0.05); B and C (*p* < 0.05).

### Comparison between upwelling and non-upwelling periods

**Figure 4:**
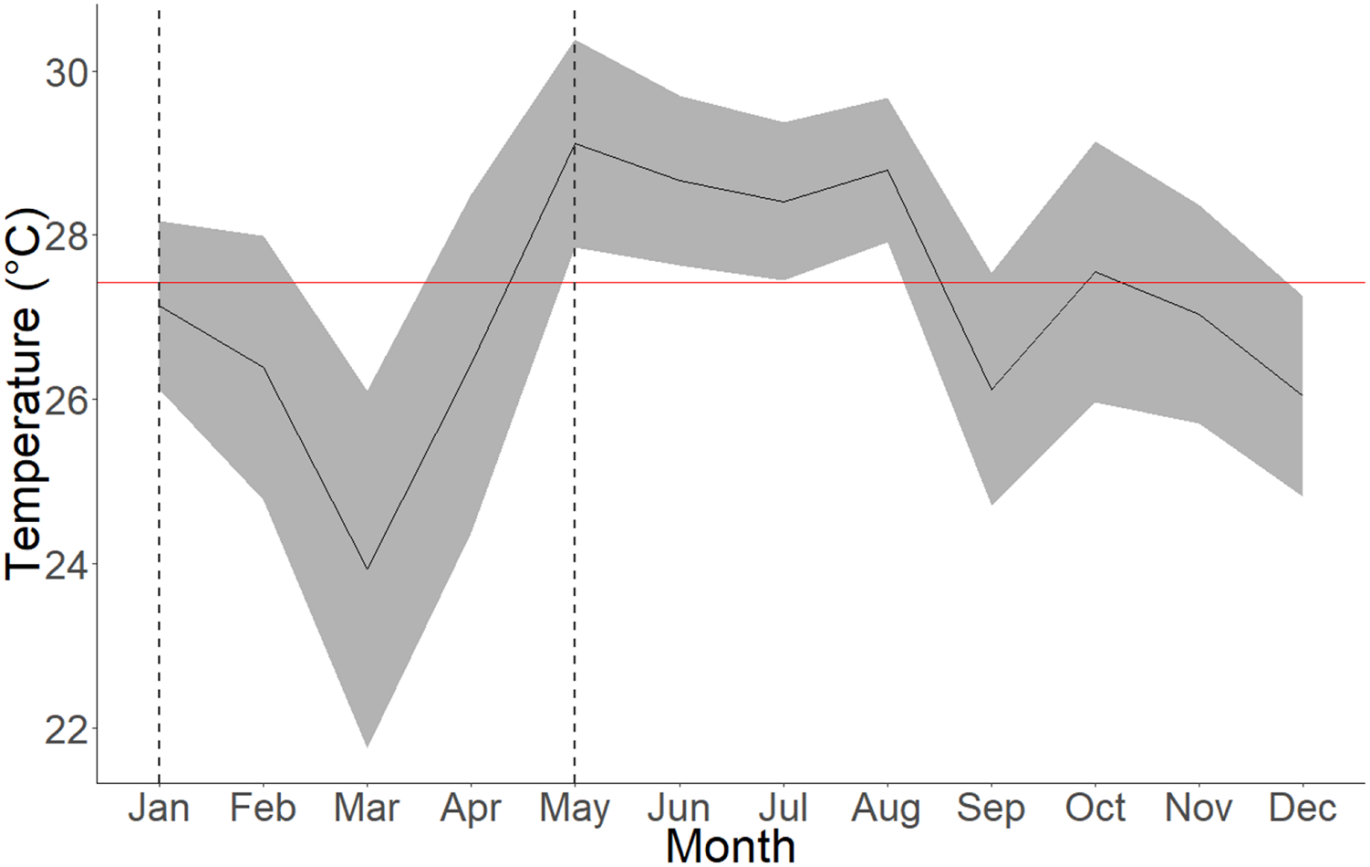
Average seawater temperatures in Culebra Bay over the year 2020. Gray lines represent standard deviations, red line represents 2020 mean temperature and area between dashed lines represent the upwelling period. The mean temperature for the upwelling period (January to April) is 25.97 ± 2.16 °C (min = 18.53 °C; max = 29.91 °C) and 27.92 ± 1.62 °C (min = 22.2 °C; max = 31.08 °C) for the non-upwelling period (May to December).

Coral growth is significantly impacted by seasonal upwelling for the 2 and 5 cm initial class size. However, no significant difference between periods was observed for 8 cm coral fragments (Table 3). Regardless of the initial size, *Pocillopora* fragments grow 48% faster on average during the non-upwelling season, coinciding with a higher mean temperature during this period (Fig. 4).

**Table 3:** Comparison of month relative length means difference in growth between upwelling season (January to April) and non-upwelling season (May to December).

	Length (cm month^−1^)		Width (cm month ^−1^)		Area (cm² month^−1^)	
Initial class size	Upwelling	No upwelling	Upwelling	No upwelling	Upwelling	No upwelling
A	0.27 ± 0.12	0.39 ± 0.47*	0.19 ± 0.10	0.35 ± 0.25*	0.60 ± 0.39	2.3 ± 2.24*
B	0.32 ± 0.17	0.49 ± 0.82*	0.31 ± 0.02	0.48 ± 0.83*	2.08 ± 0.64	4.85 ± 8.84*
C	0.22 ± 0.03	0.32 ± 0.51	0.27 ± 0.06	0.28 ± 0.44	3.22 ± 0.58	3.22 ± 3.99
Total	0.27 ± 0.60	0.4 ± 0.59*	0.26 ± 0.08	0.37 ± 0.54*	1.97 ± 1.23	3.46 ± 3.99*

**Note:**

T-tests show significant difference of mean growth (represented by “*” in the table) between the upwelling season and the non-upwelling season for all initial class sizes (A = 2 cm, B = 5 cm and C = 8 cm). Total: A (t = −5.8; *p* < 0.001; df = 57), B (t = −6.02; *p* < 0.001; df = 57) and C (t = −6.93; *p* < 0.001; df = 57).

## Discussion

Coral reef management is a key issue in the current context of global change. Assessing the resilience of coral species and identifying sites conducive to the survival of corals is thus crucial in order to improve management actions (McLeod et al., 2021). While whether corals will have the ability to acclimate rapidly enough to the new environmental conditions is still under debate (Maynard et al., 2008; Eakin, 2014; Torda et al., 2017; Coles et al., 2018), active coral reef restoration is emerging worldwide as a tool for assisting coral reef recovery and rehabilitation (Rinkevich, 2019). Several restoration strategies have been developed, such as structural complexity enhancement by artificial substrates, which increase coral recruitment and can be used as an alternative or addition to coral transplantation for reef restoration purposes (Yanovski & Abelson, 2019; Hein et al., 2020). This type of structures has mainly been used in the Indo-Pacific, specifically in the Maldives and Thailand, where metallic structures called “frames” were set up (Hein, Couture & Scott, 2018; Hein et al., 2020). However, their use is recent, the coral species used are not the same, and environmental conditions in those areas are different from those in the ETP (Kench, 2009; Lizano & Alfaro, 2014). This makes comparisons difficult and obtained data are not necessarily transferable to other reefs in other oceanic regions (Sherman, Gilliam & Spieler, 2001). Therefore, it appears necessary to generate data on the performance of this kind of structures under the conditions in the ETP, in order to assess their viability in this oceanic region.

Evaluating this strategy involves monitoring fragment mortality and growth, and associating the data with environmental information from the area. In this study, *Pocillopora* fragment mortality was not significantly influenced by initial fragment size. The month with the highest mortality was February, just one month after the fragmentation event and start of the experiment, with nine dead fragments, 66% of which were 2 cm long. A positive relationship between coral fragment survival and size has been established in several studies (Connell, 1973; Hughes, 1984; Lizcano-Sandoval, Londoño-Cruz & Zapata, 2018; Ishida-Catañeda et al., 2020). Research on *Pocillopora* has found that smaller coral fragments are more vulnerable to detrimental factors due to their greater surface/volume ratio. This means that a lesion on the coral tissue can cause greater damage than in larger fragments, and thus it makes them more sensitive to manipulation, competition with other organisms and predation (Raymundo & Maypa, 2004; Lizcano-Sandoval, Londoño-Cruz & Zapata, 2018; Ishida-Catañeda et al., 2020). According to the micro fragmentation theory, this can be compensated by small fragments growing more rapidly at first compared to larger fragments or colonies, so that they can quickly reach a size which makes them less vulnerable to impacts (Forsman, Rinkenvich & Hunter, 2006; Page, Muller & Vaughan, 2018; Tortolero-Langarica et al., 2020). However, our results show no significant differences between linear growth rates of 2 cm fragments and the other class sizes. The bay possesses a great productivity (Fernández-García, 2007; Stuhldreier et al., 2015a) making the structures a suitable substrate for the settlement of benthic, fast-growing, opportunistic species, such as barnacles, ascidians, and sponges. These benthic organisms compete with coral fragments and can affect their growth and survival (Glynn et al., 2017). Although monthly maintenance of the structures limits this effect, their great abundance and presence on the “spiders” could have had an effect on coral fragments, especially on the smaller class size, because of their limited surface. These smaller coral fragments might not have been able to compete for space against these other organisms. Due to their small size, several of their energy reserves were probably not available, and therefore were presumably being used to recover from fragmentation stress, and not for growth and defense against competitors (Leuzinger, Anthony & Williw, 2003; Henry & Hart, 2005). Hence, it is assumed that the small fragments used in this study were the most fragile and affected by these detrimental factors, and thus did not resist the stress of fragmentation and change in environment during the first weeks of the experiment.

The loss of 32 coral fragments during the course of the experiment could be explained by several reasons: (i) cable ties being either too tight and resulting in fragment break, or too loose cable ties, causing them to fall, especially small (2 cm) fragments; or (ii) clumsiness during the manual cleaning and maintenance of the restoration structures. These lost fragments are not included in the survivorship results, since it is not possible to determine whether they survived in the reef or not. The observed decrease in growth and fragment size between two consecutive months in some coral fragments can possibly be a result of intrinsic variations of the colony, either by partial mortality of coral tissue, or natural fragmentation processes.

The growth rates of *P. damicornis* and *P. elegans* in Culebra Bay were determined in 1995–1996, with a mean of 5.3 ± 0.4 cm year^−1^ and 4.1 ± 0.6 cm year^−1^, respectively, using 13 cm long fragments (Jiménez & Cortés, 2003). Under stressful conditions, in the presence of the competitor macroalgae C*aulerpa sertularioides*, *Pocillopora* corals (no initial size reported) in the bay show a lower growth rate (2.5 cm year^−1^) than without it (4.2 cm year^−1^) (Fernández-García, 2007). In the present study, a length growth rate of 4.49 ± 1.19 cm year^−1^, 5.35 ± 1.48 cm year^−1^ and 3.25 ± 2.22 cm year^−1^ respectively for the 2, 5 and 8 cm initial size was established (Table 2), along with and an overall growth rate of 4.12 ± 2.77 cm year^−1^. These results seem to follow the rate calculated by Jiménez & Cortés (2003) on the reef in the 1990s, when coral reef ecosystems in the bay were considered healthier. This means that fragments on the “spiders” are growing at a similar rate to corals growing naturally in the reef. These results are quite high compared to other reefs of the ETP: for example, in Caño Island (South Pacific of Costa Rica), the rate for 15–25 cm long fragments is 2.9 ± 0.3 cm year^−1^ for *P. damicornis* and 3.17 ± 0.3 cm year^−1^ for *P. elegans* (Guzmán & Cortés, 1989). In the Central Mexican Pacific, the growth rate is 3.5 ± 0.6 cm year^−1^, with no initial size being mentioned (Tortolero-Langarica et al., 2017). The lowest *Pocillopora* growth rates reported for the ETP are in the Gulf of Chiquiri (Panama) and Colombia, with 2.6 cm year^−1^ (initial size = 6.3 ± 1.4 cm) (Randall et al., 2020) and 2.3 cm year^−1^ (no initial size mentioned) (Zapata & Vargas-Angel, 2003), respectively. It is hypothesized that the higher growth rate in Culebra Bay is linked to the specific conditions of the bay, with the seasonal upwelling bringing up more productive waters, which could lead to an increase of the corals heterotrophic feeding (Jiménez & Cortés, 2003). These results also show that the corals of Culebra Bay are particularly acclimated to the specific environmental conditions of the bay, which make them an example of growth under suboptimal conditions, with incursions of colder and more acidic waters (Rixen, Jiménez & Cortés, 2012; Sánchez-Noguera et al., 2018).

Understanding the best initial fragment size is vital for efficient restoration activities. Even though this has been established for many species in other regions, information on *Pocillopora* corals and under ETP conditions is limited (but see Lizcano-Sandoval, Londoño-Cruz & Zapata, 2018; Ishida-Catañeda et al., 2020). Moreover, even though initial fragment size seems to be an important factor for coral growth, most studies do not consider it in their analysis. Based on our results, it can be assumed that 2 cm fragments are not of an optimal size when rearing as many corals as possible onto the reef, since they experience high mortality during the first months after fragmentation, and are more fragile and prone to breaking. On the other hand, it was found that larger fragments (between 5 and 8 cm) grow at a similar rate while experiencing lower mortality. However, extracting larger fragments and repeated fragmentation of coral colonies can compromise the survival of these donor colonies, and it may lead to reduced sexual reproduction (Zakai, Levy & Chadwick-Furman, 2000), which could in turn impact the development of the whole coral reef ecosystem. The recovery of donor colonies after fragmentation events should also be assessed in order to evaluate the impact of extracting large coral fragments.

Corals from Culebra Bay have already been confronted by stressing episodes which have had an effect on the coral reef ecosystem (Jiménez, 2001; Alvarado, Cortés & Reyes-Bonilla, 2012; Fernández-García et al., 2012). Nonetheless, ETP reefs have shown a high resilience to stressing events (Romero-Torres et al., 2020), which would allow large-scale rehabilitation even after severe disturbances, such as El Niño events (Williams et al., 2018). Culebra Bay is located in one of the three seasonal upwelling areas of the ETP, which from December to April brings colder and more acidic waters to the surface, with a higher concentration of nutrients (Rixen, Jiménez & Cortés, 2012; Stuhldreier et al., 2015a; Sánchez-Noguera et al., 2018). The decrease in seawater temperature and increase in productivity can have an effect on coral growth and survival (Clausen & Roth, 1975; Coles & Jokiel, 1978). Our results show a difference in growth between the upwelling and non-upwelling periods: coral growth increased 48% on average during the non-upwelling period (May to December) compared to the upwelling period. Similar results were obtained in the bay when comparing the growth rate of *Pocillopora* spp. during seasons, with higher rates occurring during the non-upwelling season (Fernández-García, 2007). However, 8 cm fragments were not found to be significantly impacted by the presence of the seasonal upwelling. These fragments also correspond to the size class with the lowest growth rate. It is thus hypothesised that since these coral fragments are already large, they allocate less energy to their growth rather than in other physiological processes. The lower temperatures during upwelling, with incursions of 18.5 °C waters, could also be responsible for the higher mortality of coral fragments during the first months of the experiment, coinciding with the possible stress caused by fragmentation and smaller size of the coral fragments. Studies on the effect of cold water on branching corals have found that in the short term, low temperatures can be more damaging than warm temperatures, but acclimatation is possible after a few weeks, and corals can recover quickly when temperatures rise back (Jokiel & Coles, 1977; Roth, Goericke & Deheyn, 2012; Rodríguez-Troncoso et al., 2014). Even though it has been suggested that in case of stress, corals preferentially use heterotrophic feeding and use lipids stored in their tissues (Grottoli, Rodrigues & Juarez, 2004; Grottoli, Rodrigues & Palardy, 2016; Rodríguez-Troncoso, Carpizo-Ituarte & Cupul-Magaña, 2010), it seems that this type of feeding is less efficient in terms of nutrition than autotrophy. Considering this, we suggest that restoration activities in Culebra Bay, such as fragmentation of new corals, should take place after the upwelling season; thus, smaller newly generated fragments would have higher chances of surviving the initial months and could reach a larger size before the upwelling begun and temperatures decreased again. Restoration efforts in these areas of the ETP where seasonal upwelling is present should thus take into account these considerations for the optimal growth of *Pocillopora* spp. fragments.

Considering our results, the use of “spiders” is a viable option for coral reef rehabilitation and restoration, and their effect could be scaled up by increasing the number of structures in order to cover a greater extension and add more structural complexity to the reef. Even though 47.61% of initial coral fragments were either lost or died during the experiment, it allowed us to determine the most resistant fragment sizes, and thus those that should be used on future restoration efforts. Other factors must also be considered, such as the location of the structures and the donor sites for coral fragments. Beyond the technical aspects of a restoration project, two main limiting factors exist: the economic aspect—which includes the costs of setting up and maintaining the structures (Dunning, 2015; Hesley et al., 2017)—and the communication about conservation strategy (Dunning, 2015). These structures have a relatively lower cost to other underwater coral nurseries, only costing around US$25 per structure (US$0.66 per coral fragment, excluding indirect costs). Moreover, they require less time to clean and maintain: one “spider” can be cleaned by one diver in around 15 min, which is considerably less time than what is needed for other structures in the same restoration project, such as rope nurseries or PVC and glass fiber coral trees (1.6 m long × 1.2 m wide) (S. Fabregat-Malé, 2021, personal communication). These limitations can be bypassed with the involvement of local communities and tourists by creating a participative program (Hein et al., 2019) in which the cost of the project will be reduced and there will be an increase in public awareness and workforce, allowing for larger-scale restoration efforts. The restoration project in Culebra Bay, which started on August 2019 with coral gardening techniques (S. Fabregat-Malé et al., 2020, unpublished data), is now complemented by the use of artificial structures in this project, leading towards its expansion through greater restoration efforts and the implementation of a participatory program. This study complements those already carried out and in progress, allowing an improvement of the techniques used to optimise the restoration efforts of reefs in Culebra Bay.

## Conclusions

Active restoration has become a key management tool to rehabilitate anthropogenically deteriorated coral reefs. In Culebra Bay, North Pacific of Costa Rica, coral reefs have suffered several degrading episodes in the last decades but are currently subject to ecological restoration actions. Various transplantation techniques are used with the genus *Pocillopora*, including the coral gardening approach. Here, a new technique in the ETP was tested, consisting in rearing coral fragments on artificial structures (“spiders”), which not only work as a nursery and substrate for coral fragments to grow on, but also add structural complexity to the reef. Our findings show that small *Pocillopora* fragments are especially vulnerable and sensitive to environmental stresses during the first months after fragmentation, which results in higher mortality rates. Even though we found no significant differences in linear growth between size classes, the smallest class size appears to be less optimal than larger ones if restoration efforts are to be scaled. The presence of a seasonal upwelling in the bay has an effect on coral growth, most likely due to cold temperatures. The upwelling brings up deeper and nutrient-rich waters, resulting in the proliferation of opportunistic and highly competitive benthic organisms (Fernández-García et al., 2012; Stuhldreier et al., 2015b), which could potentially have an effect on coral growth and survival. This information is key in order to plan restoration activities in areas affected by seasonal upwelling, since fragments will grow more optimally if transplanted at the end of the upwelling season, and will be robust enough to cope with the next upwelling period as they will have reached a larger size. Our data also show how corals can survive under suboptimal conditions when acclimated to such an environment. Studying the particular characteristics of these areas is essential to understanding, optimising and innovating reef restoration strategies at local scales, especially in the ETP region, where information is still scarce.

## Supplemental Information

10.7717/peerj.13248/supp-1 Supplemental Information 1 Growth fragments monitoring.

10.7717/peerj.13248/supp-2 Supplemental Information 2 Sea water temperatures 2020.

## Additional Information and Declarations

### Data Availability

The following information was supplied regarding data availability: The raw data is available in the Supplemental Files. The code is available on RPubs by R Studio: https://rpubs.com/LisaCom/806915.
